# Evaluating the effect of ursodeoxycholic acid (UDCA) in comparison with dexamethasone and diclofenac in a rat model of rheumatoid arthritis

**DOI:** 10.25122/jml-2023-0107

**Published:** 2023-10

**Authors:** Yamama Raad Abduljaleel, Ahmed Hamed Jwaid, Aseel Kamil Hussein

**Affiliations:** 1 Department of Pharmacology and Toxicology, College of Pharmacy, University of Baghdad, Baghdad, Iraq; 2 College of Veterinary Medicine, University of Baghdad, Baghdad, Iraq

**Keywords:** Complete Freund adjuvant (CFA), rheumatoid arthritis (RA), ursodeoxycholic acid (UDCA)

## Abstract

Ursodeoxycholic acid (UDCA) is known for its major effects on the liver, but its impact on autoimmune diseases is not well understood. This study aimed to assess the effectiveness of UDCA in controlling rheumatoid arthritis (RA) in an *in vivo* setting. Experimental RA was induced in rats using Freund's complete adjuvant, and the effects of UDCA (50,100 mg/kg) were compared to those of dexamethasone and diclofenac by measuring changes in paw size, IL-17, pro-inflammatory cytokines, oxidative stress (GSH, MDA), and radiological changes. The administration of UDCA resulted in decreased cartilage damage, reduced paw edema, and a decrease in the release of pro-inflammatory cytokines and oxidative stress. Additionally, X-ray joint alterations were observed in the UDCA-treated group compared to the dexamethasone and diclofenac groups. These results suggest that UDCA has anti-rheumatoid arthritis properties due to its ability to minimize oxidative stress and inflammation in arthritis-affected rats.

## INTRODUCTION

The autoimmune disease rheumatoid arthritis (RA) is a chronic condition that primarily affects the joints but can also affect other organs and tissues. Inflammation of the synovial lining of joints leads to discomfort, stiffness, and edema, which, over time, can destroy bone and cartilage within the joint [1]. As a systemic disease, RA may affect other parts of the body, including blood vessels, lungs, skin, and eyes, and it affects the same joints on both sides of the body [2]. With no current cure, RA is a long-lasting condition that is estimated to affect approximately 0.24% of people worldwide, or approximately 23 million people [3].

Pro-inflammatory cytokines, including interleukin-6 (IL-6), interleukin-1β (IL-1β) [4], and tumor necrosis factor-alpha (TNF-α) [5, 6], significantly influence RA pathogenesis. These cytokines increase the recruitment of immune cells to the area of inflammation, promote the production of enzymes that degrade joint tissue, and promote the proliferation of synovial cells. These processes contribute to the formation of pannus, exacerbating the influx of immune cells to the site of inflammation and ultimately advancing the progression of various diseases, including non-alcoholic fatty liver disease (NAFLD) and other related conditions [7]. Interleukin 17 (IL-17), a cytokine produced by Th17 cells, plays a critical role in the onset of RA [8, 9]. It promotes pannus development and joint degeneration by promoting osteoclastogenesis and synovial angiogenesis [10, 11].

Several treatments are available to help manage the disease and improve symptoms. Disease-modifying anti-rheumatic medications (DMARDs), including sulfasalazine, hydroxychloroquine, methotrexate, and leflunomide, are often used to stop joint damage and reduce the progression of RA [12]. These medications work by decreasing inflammation and targeting the immune system, but they can take weeks or months to take full effect. Steroids and non-steroidal anti-inflammatory drugs (NSAIDs) are also commonly used to treat RA, with NSAIDs helping to reduce inflammation and pain and steroids helping to reduce inflammation and relieve symptoms quickly [13, 14]. By blocking the action of these cytokines, medications can reduce inflammation, slow or halt joint damage, and provide symptomatic relief for patients with RA.

Despite the effectiveness of the above treatments for RA, their long-term use is limited due to unavoidable but serious adverse effects, such as liver and bone marrow damage, heart problems, and stomach discomfort [15]. Genetic research on RA has led to the development of novel, better-tolerated medications called biologics, such as infliximab, rituximab, sarilumab, tocilizumab, and tofacitinib. These drugs have the potential to target immune system components that lead to inflammation-induced joint and tissue damage [16]. However, several investigations have shown that individuals taking these biologics are more susceptible to infections of the genitourinary system, skin, soft tissue, and joints than those taking conventional medications [17]. As no drug has been effective in reducing the effects of RA up until now, it is desirable to develop new drugs that can overcome these limitations.

Ursodeoxycholic acid (UDCA), also known as ursodiol (USAN), is a secondary bile acid produced in the liver from primary bile acids such as chenodeoxycholic acid (CDCA). UDCA can also be produced by certain bacteria in the intestines through a process called bacterial 7 dehydroxylation. UDCA constitutes approximately 5% of the total bile acids in humans and is known for its hepatoprotective and choleretic properties. It is used to treat many hepatic illnesses, including primary biliary cholangitis (PBC), a chronic autoimmune liver condition characterized by the gradual destruction of the liver's tiny bile ducts [18, 19]. However, there are few findings on UDCA's impact on autoimmune disorders, such as autoimmune hepatitis. UDCA was used in an attempt to treat autoimmune hepatitis, but no significant results were observed [20]. Nevertheless, it prevented dendritic cells from activating via the farnesoid X receptor, which reduced eosinophil-mediated inflammation [21], as evidenced by the interaction between dendritic cells and T cells and its impact on T cell performance. Recent investigations have suggested that UDCA may function as an immunomodulator in autoimmune diseases. Due to its ability to reduce monocyte expression of TNF-induced IL-8, UDCA may have therapeutic potential in inflammatory diseases [22]. These studies suggest that UDCA may have the potential to treat autoimmune diseases, including RA. In this study, we investigated the effects of UDCA treatment on RA.

## MATERIAL AND METHODS

### Drugs and chemicals

Freund's Complete Adjuvant (FCA) was obtained from Sigma Aldrich (USA). ELISA kits for IL-17, GSH, and MDA were obtained from Pars Biochem in China. Diclofenac 50 mg was obtained from Novartis (Switzerland), and dexamethasone powder in bicarbonate vehicle 5 ml/0.5 mg was obtained from SDI (Iraq). Sodium bicarbonate was supplied by an Iraqi company. Ursodeoxycholic acid (UDCA) was purchased from Picasso (China). All other compounds used were of the highest purity and analytical grade.

### Animals

Thirty-six adult Wistar Albino rats of both sexes, weighing 180-230 g, were used in the experiment. The rats were obtained from the Pharmacy College at the University of Baghdad and kept under normal conditions with complete access to a normal diet and water and suitable temperature, humidity, and light/dark cycles [23].

### Preparation of UDCA solution

A total of 100 g of chemical-grade sodium bicarbonate powder and 30 g of UDCA were accurately weighed. Approximately 1800 ml of hot sterile water was added to a 2,500 ml volumetric flask placed on a moving hot plate, and sodium bicarbonate was gradually added to the mixture. After the complete solubilization of the sodium bicarbonate, the mixture was brought to a rapid boil. Next, 25% (7.5 g) of the total UDCA volume was added to the boiling sodium bicarbonate solution. The remaining UDCA was added to the solution at 10-minute intervals, gradually increasing until complete dissolution was achieved, which took about 30 minutes. The solution was diluted to 2,000 ml using hot, sterile water. Finally, the finished solution was poured into amber containers and stored at 4°C in the refrigerator [24].

### Freund's complete adjuvant-induced arthritis

FCA is a specific type of adjuvant used to enhance the immune response to an antigen. It contains a concentration of 10 mg/ml of inactivated, heat-killed *Mycobacterium tuberculosis* bacteria suspended in liquid paraffin. All rats except those in the control group were intradermally injected with 0.1 ml of FCA into the left hind paw to induce arthritis. On the same day and the following day, a 0.1 cc booster subcutaneous injection was administered around the tail to further enhance the immune response to FCA [25, 26]. A 7-day interval following the arthritis induction was allowed to ensure the development of arthritis among the rats.

### Experimental design

We divided thirty-six rats into six equal groups. One group served as a control without inducing rheumatoid arthritis, while the others underwent arthritis induction. To induce arthritis, rats were anesthetized using diethyl ether. A cotton ball with 2.75 ml/l of diethyl ether was placed in a clear glass desiccator to minimize skin irritation during anesthesia. The rats were then injected with FCA intradermally at the medial part of the ankle [27].

**Negative control group (N=6) (normal):** This group received 5% sodium bicarbonate orally for 21 days. Sodium bicarbonate was the vehicle for all test groups except for the positive control group.

**Positive control group (N=6) (induction group):** Rats in this group were observed for the entire 21-day study period without additional interventions after rheumatoid arthritis induction.

**Dexamethasone group (N=6):** Rats in this group were administered dexamethasone orally (in bicarbonate solution) on the same day as the FCA injection. The dose of dexamethasone was 0.5 mg/kg, and treatment continued for 21 days [28].

**Diclofenac group (N=6):** Rats in this group received diclofenac orally (in bicarbonate solution) on the same day as the FCA injection. The dose of diclofenac administered was 5 mg/kg, and this treatment also continued for 21 days [29].

**100 mg UDCA group (N=6):** In this group, rats were given UDCA orally (in bicarbonate solution) on the same day as the FCA injection. The dose of UDCA administered was 100 mg/kg, and the treatment continued for 21 days [30].

**50 mg UDCA group (N=6):** Similar to the previous group, this group of rats received UDCA orally (in a bicarbonate solution) on the same day as the FCA injection. However, the dose of UDCA administered in this group was 50 mg/kg for 21 days [30].

### Paw edema

**Figure 1:**
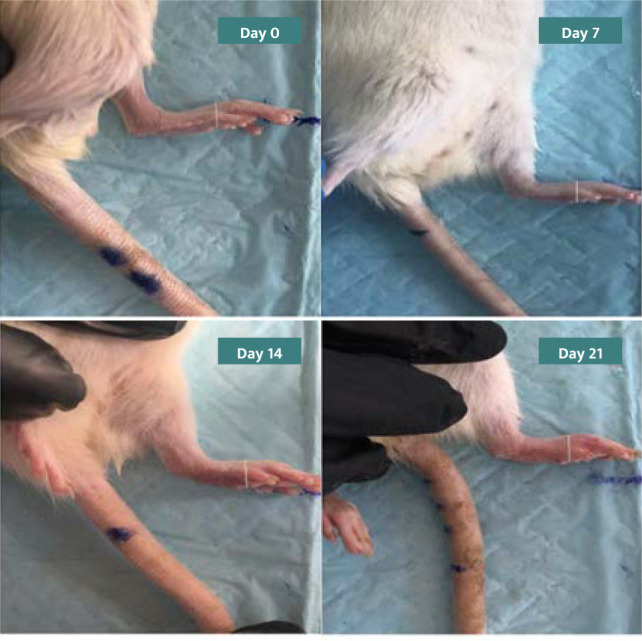
Paw size measurement by vernia

The thickness of the hind limb's paw was initially measured the day after the administration of FCA injection, and subsequent measurements were taken at seven-day intervals up to twenty-one days. The measurement at seven days provided data on the initial lesions, while the measurement at fourteen days was essential to determine secondary lesions (Figure 1) [31].

### Biochemical analysis

After euthanizing the animals using diethyl ether [32], blood samples were collected via heart puncture at the end of the research, just before the cervical dislocation of the animals. These samples were immediately transferred into gel tubes and left for 30 minutes to allow clot formation. Following a 30-minute centrifugation to achieve clear serum, blood sample temperatures were kept at -20 °C until the day of analysis. Stored sera were utilized to estimate serum activities of IL-17, intracellular glutathione (GSH), and malondialdehyde (MDA). This analysis was performed using the sandwich qualitative enzyme-linked immunoassay (ELISA) technique [33, 34].

### Radiological changes

The hind limbs were radiographed using a portable Siemens x-ray machine with settings at KV 40, MS 0.5, and a diaphragm height of 70 cm. A radiological assessment was conducted to evaluate both the joint space and its associated damage and soft tissue edema. These radiological findings were then translated into numerical data [35].

### Statistical analysis

Statistical analysis was performed using GraphPad Prism 5 software (GraphPad Software Inc., La Jolla, USA) [35]. Descriptive statistics and one-way ANOVA with a multiple comparison test following Bonferroni corrections (with an alpha level set at 0.05) were conducted. In cases where the data did not meet the criteria for a Gaussian distribution, the Kruskal-Wallis test with Dunns post-multiple comparison test (at a significance level of 95%) was employed for statistical analysis.

## RESULTS

### Paw edema

**Figure 2:**
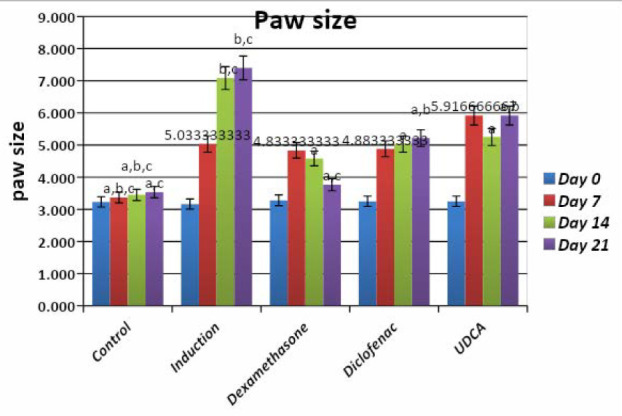
Effect of UDCA 100 mg/kg on paw size compared to dexamethasone and diclofenac Each value represents means±standard error of means (SEM) (N=6/group). Values expressed in non-identical small letters (a, b, c) are significantly different (p<0.05). a-represent significant difference with the induction group; b-represent significant difference with dexamethasone group; c-represent significant difference with the diclofenac group.

**Figure 3:**
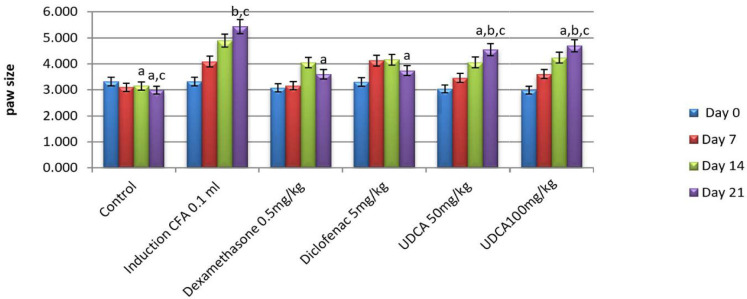
Effect of UDCA 50mg/kg on paw size compared to dexamethasone and diclofenac Each value represents means±standard error of means (SEM) (N=6/group). Values expressed in non-identical small letters (a, b, c) are significantly different (p<0.05). a-represent significant difference with the induction group; b-represent significant difference with dexamethasone group; c-represent significant difference with the diclofenac group.

The paw thickness significantly increased following the induction of arthritis. However, a gradual decrease was observed after the application of treatment. Although all groups showed a significant decrease in the thickness of the paw after treatment, the dexamethasone group showed the best results, followed by the diclofenac and the UDCA group (Tables 1,2 and Figures 2, 3).

**Table 1:** Comparative analysis of the effect of UDCA (100 mg/kg) on paw edema (mm measured by digital vernier) compared to dexamethasone and diclofenac

Groups	Day 0	Day 7	Day 14	Day 21
Normal control	3.23±0.09	3.37±0.1a,b,c	3.45±0.09a,b,c	3.53±0.02a,c
FCA (0.1 ml FCA footpad) (induction group)	3.17±0.09	5.03±0.13	7.08±0.1b,c	7.4±0.30b,c 666
FCA+ Dexamethasone (0.5 mg/kg)	3.28±0.1	4.83±0.27	4.58±0.24a	3.77±0.16a,c
FCA+ Diclofenac (5 mg/kg)	3.25±0.09	4.88±0.27	5.03±0.22a	5.21±0.16a,b
FCA+ UDCA (100 mg/kg)	3.25±0.09	5.91±0.37	5.25±0.25a	5.91±0.24a,b

Each value represents means±standard error of means (SEM) (N=6/group). Values expressed in non-identical small letters (a, b, c) are significantly different (p<0.05). a-represent significant difference with the induction group; b-represent significant difference with dexamethasone group; c-represent significant difference with the diclofenac group.

### Biochemical Analysis

#### Serum IL-17 levels

**Figure 4:**
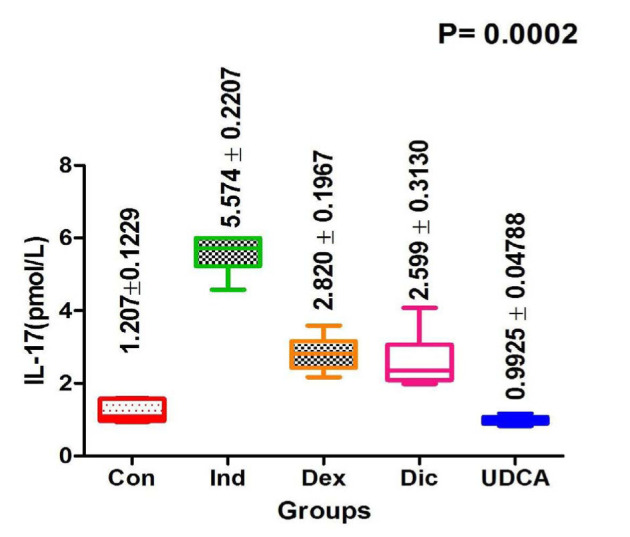
Comparison of IL-17 levels among treatment groups at 21 days post-treatment

Serum IL-17 levels were significantly higher in the induction group compared to the other groups. In the induction rats, the IL-17 level was 5.57±0.22 pmol/L, while in the UDCA group, the level of IL-17 decreased to 0.1±0.04 pmol/L, reaching normal values, as shown in Figure 4.

**Table 2:** Comparative analysis of the effect of UDCA (50 mg/kg) on paw edema (mm measured by digital vernier) compared to dexamethasone and diclofenac

Groups	Day 0	Day 7	Day 14	Day 21
Normal control	3.322±0.282	3.103±0.18	3.150±0.17a	2.985±0.08a,c
FCA (0.1 ml FCA footpad) (induction group)	3.32175±0.282	4.09±0.312	4.895±0.364	5.4375±0.208b,c
FCA+Dexamethasone (0.5 mg/kg)	3.08±0.138	3.16±0.103	4.05±0.045	3.6±0.1a
FCA+ Diclofenac (5 mg/kg)	3.3±0.198	4.132±0.202	4.16±0.14	3.74±0.07a
FCA+ UDCA (50 mg/kg)	3.04 ±0.09	3.43±0.16	4.04±0.2	4.5±0.1a,b,c
FCA+ UDCA (100 mg/kg)	2.985±0.08	3.61±0.172	4.237±0.09	4.7±0.108a,b,c

Each value represents means± standard error of means (SEM) (N=6/group). Values expressed in non-identical small letters (a, b, c) are significantly different (p<0.05). a-represents significant difference with the induction group; b-represents significant difference with the dexamethasone group; c-represents significant difference with diclofenac group.

#### Serum GSH levels

**Figure 5:**
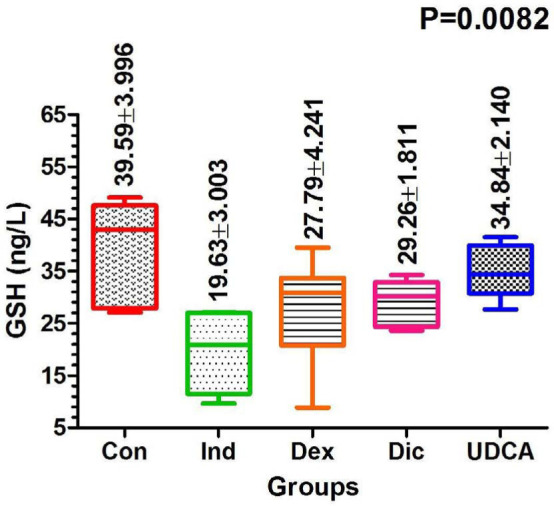
Comparison of GSH levels among treatment groups at 21 days post-treatment

Serum GSH levels were significantly lower in the induced group (19.63±3 ng/L) compared to the control group by the end of the 21-day experiment. However, the level of GSH reached 34.84 ng/L in the UDCA group by the end of the experiment (Figure 5).

#### MDA serum level

**Figure 6:**
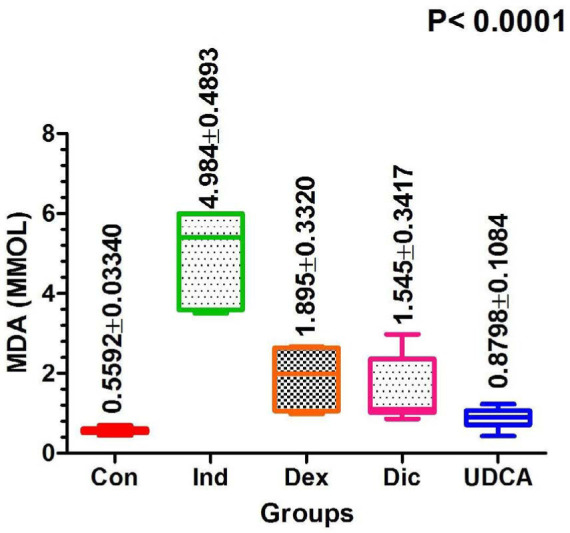
Comparison of MDA levels among treatment groups at 21 days post-treatment

MDA levels were significantly elevated in the induction group (4.98±0.49 mmol/L) on day 21. Conversely, in the UDCA group, they were significantly lower at 0.88±0.1 mmol/L (Figure 6).

### Radiological changes

**Figure 7:**
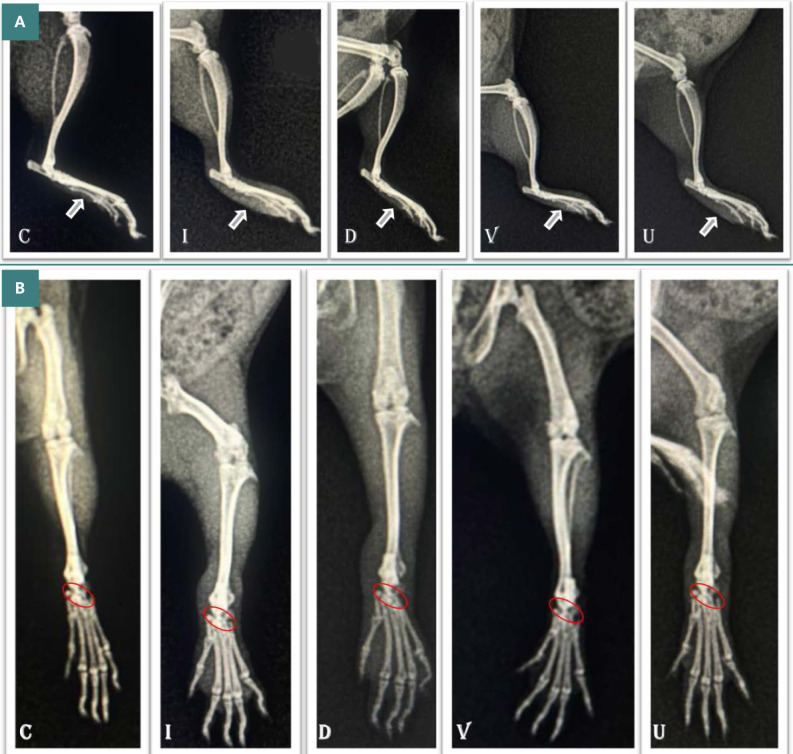
A: Limb swelling in the induced rheumatoid arthritis group (I) with the highest severity score of 3. Group U, V, and D show reduced swelling compared to Group C, with a score of 0. B: Significant changes in joint shape and space in Group I and V compared to Group U, which closely resembles the normal joint structure as seen in C.

Significant soft tissue swelling was observed in the induction group, followed by the UDCA and diclofenac groups, with the least swelling observed in the dexamethasone group (Figure 7A). On the dorso-plantar view, erosions associated with joint space narrowing and damage to the tarsal bones' structure and alignment were significantly more pronounced in the induction group. These effects were followed by the dexamethasone and diclofenac groups, with the least amount of damage observed in the UDCA group, as shown in Figure 7B.

**Figure 8:**
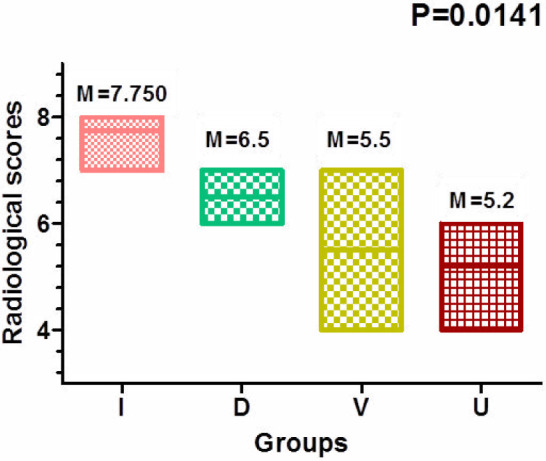
Vertical bar graph illustrating the numerical values of radiological signs based on Binder scoring, with significant differences observed between the U and I groups. (I=induction group, D=dexamethasone group, V=diclofenac group, U=UDCA 100 mg group).

When the scores of each group were calculated and compared among the groups, the lowest values were observed in the UDCA group, while the highest values were found in the untreated group (Figure 8).

## DISCUSSION

**Figure 9:**
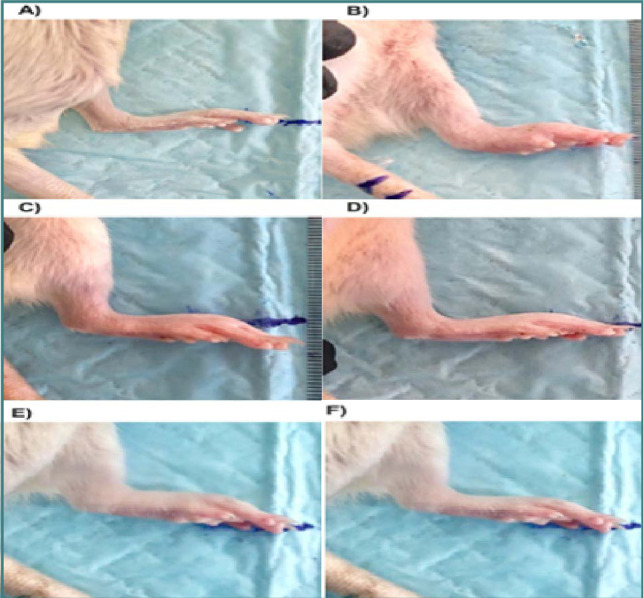
Visual changes in edema: (A) negative control (Normal) group (sodium bicarbonate 5%) at day 21; (B) positive control (FCA induction) at day 21; (C) FCA+ dexamethasone (0.5 mg/kg) at day 21; (D) FCA+ diclofenac (5mg/kg) at day 21; (E) FCA+ UDCA (50 mg/kg) at day 21(F) FCA+ UDCA (100 mg/kg) at day 21.

UDCA is known for its impact on liver conditions like primary biliary cirrhosis and jaundice [22]. O'Dwyer *et al*. discovered that UDCA lowers the release of pro-inflammatory cytokines and chemotactic factors and contains anti-atherogenic and anti-inflammatory properties [36, 37]. However, the specific mechanism behind the anti-inflammatory activity of UDCA is not yet fully understood. In this study, we demonstrated the effectiveness of UDCA compared with dexamethasone and diclofenac as a treatment for arthritis in a rat model induced by Freund's complete adjuvant (FCA). FCA is commonly used in preclinical research on the etiology of rheumatoid arthritis and to test potential therapeutic interventions [38]. The effectiveness of therapeutic medications in this model is substantially associated with human rheumatoid arthritis, as rapid erosive illness is a hallmark of this condition [39]. Bacterial peptidoglycan and muramyl dipeptide induce arthritis in adjuvant arthritis. Mycobacteria and cartilage proteoglycans have structural similarities in rats, resulting in cell-mediated autoimmunity. Rats were selected as the experimental subjects for this arthritis study due to their tendency to develop persistent joint inflammation, involvement of inflammatory cells, erosion of joint cartilage, and bone degradation [40]. To cause arthritis in the rats, we used FCA, and inflammation was induced on day 3, peaking on day 14. Following the FCA injection, the rats' hind paws exhibited noticeable swelling and hyperalgesia, while the contralateral paw remained unaffected. This reaction is typically categorized as a primary response. Additionally, a delayed hypersensitivity reaction, sometimes referred to as a latent secondary inflammatory response, was observed, resulting in swelling of the tibiotarsal joint on the opposing paw a few days later. The release of bradykinin, prostaglandins, and kinins, along with the migration of leukocytes, may be the cause of the secondary reaction [40]. Our analysis and results showed that UDCA (100 mg/kg) had more prominent and consistent anti-inflammatory activity than dexamethasone and diclofenac, reducing the progression of the chronic joint swelling caused by FCA in both paws (Figure 9). In the current study, UDCA significantly lowered IL-17 and restored it nearly to normal levels compared with dexamethasone and diclofenac.

An extremely interesting and interdependent aspect of RA is oxidative stress and inflammation. Several studies have demonstrated that individuals with RA experience significant levels of oxidative stress as well as elevated inflammation [41, 42]. Numerous oxidative stress indicators, which cause inflammation, are characterized by enzymatic and non-enzymatic indicators. Understanding these indicators would help us better understand how to use UDCA to combat RA, which has shown promise in raising GSH activity and reducing MDA levels compared to other treatments [43]. This effect was associated with a decrease in lipid oxidation in chondrocyte and fibrocyte membranes [44]. In the current study, UDCA demonstrated the best outcomes, showing the least bone damage and minimal joint impact compared to the other groups. These findings align with previous research, such as the treatment of joints with FA-PPLNPs/Mtx, which also demonstrated a reduction in bone destruction [45]. Although the radiological and morphometric methods of the limb width showed a significant reduction in the dexamethasone group compared to UDCA, the accumulated numerical data performed better in UDCA compared to the affected limb (M=7.5) (Figure 3-4) with a value of M=5.2. This was already confirmed by Binder and his team in 1999 [35], when they described that the least bone erosions and least joint space narrowing, in addition to no or less alteration of the tarsal bone's structure and alignment, indicated positive progress of the treatment method.

## CONCLUSION

Based on the findings of this study, it can be concluded that UDCA at a dose of 100 mg/kg has anti-rheumatic activity comparable to that of dexamethasone and diclofenac. The anti-rheumatoid activity of UDCA is attributed to its antioxidant and anti-inflammatory properties, which inhibit the production of IL-17 and MDA lipid peroxidation. It is likely that the mechanism of action involves an increase in the antioxidant enzyme GSH. Therefore, in future studies, higher doses of UDCA and longer treatment times could be considered to improve both local and systemic symptoms of RA. These findings suggest that UDCA could be further evaluated as a potential replacement therapy for rheumatoid arthritis.
